# Preventive Effects of Dulaglutide on Disuse Muscle Atrophy Through Inhibition of Inflammation and Apoptosis by Induction of Hsp72 Expression

**DOI:** 10.3389/fphar.2020.00090

**Published:** 2020-02-21

**Authors:** Tram Thi Ngoc Nguyen, Hojung Choi, Hee-Sook Jun

**Affiliations:** ^1^ College of Pharmacy and Gachon Institute of Pharmaceutical Science, Gachon University, Incheon, South Korea; ^2^ Lee Gil Ya Cancer and Diabetes Institute, Gachon University, Incheon, South Korea; ^3^ Gachon Medical Research Institute, Gil Hospital, Incheon, South Korea

**Keywords:** disuse muscle atrophy, atrophic protein, glucagon-like peptide-1 receptor agonist, inflammatory cytokine, heat shock protein 72

## Abstract

Pathological conditions such as joint immobilization, long-time bed rest, or inactivity may result in disuse-induced muscle wasting and dysfunction. To investigate the effect of dulaglutide, a long-acting glucagon-like peptide-1 receptor agonist, on disuse muscle atrophy, disuse condition was induced by spiral wire immobilization in C57BL/6 mice and the mice were treated with dulaglutide. Dulaglutide treatment effectively improved muscle function and increased muscle mass compared with vehicle treatment. Dulaglutide inhibited the decrease of muscle fiber size and the expression of atrophic factors such as myostatin, atrogin-1/MAFbx, and muscle RING-finger protein-1 in immobilized mice. In addition, dulaglutide inhibited nuclear factor kappa B activation, leading to a decrease in the mRNA levels of proinflammatory cytokines, including tumor necrosis factor-α, interleukin (IL)-1β, and IL-6 in muscle of immobilized mice. Dulaglutide suppressed the expression of apoptotic markers such as caspase-3, cleaved poly-ADP ribose polymerase, and Bax under immobilization condition and increased the expression of heat shock protein 72 (Hsp72), which is related to the amelioration of inflammation and apoptosis during disuse time. Further study showed that dulaglutide could induce Hsp72 expression *via* the regulation of 5′-AMP-activated protein kinase signaling. Our data suggest that dulaglutide could exert beneficial effects against disuse-induced muscle atrophy.

## Introduction

Disuse-induced muscle atrophy resulting from immobilization, long-time bed rest, or inactivity not only decreases muscle mobility and quality of life but also affects overall health (Malavaki et al., 2015). In humans, 17 and 38 days of bed rest was reported to result in approximately 8% and 16% loss of calf muscle cross-sectional area (CSA) and 6% and 18% reduction in maximal voluntary knee-extension torque, respectively (Mulder et al., 2009; Rittweger et al., 2010). In general, animals subjected to numerous disuse models such as immobilization, hindlimb suspension, and denervation were shown to exhibit similar atrophic muscle characteristics, including muscle weakness associated with reduced muscle fiber size and enhanced muscle protein degradation (Jaspers and Tischler, 1984). For instance, a study with spiral wire immobilization (SWI) model reported 50% lower soleus muscle weight along with the induction of protein degradation markers after 10 days (Onda et al., 2016).

Muscle protein degradation is notably elevated in disused muscle and thought to involve the ubiquitin-proteasome-dependent pathway, caspase system pathway, and autophagy pathway (Wang et al., 2019). Of these, the ubiquitin-proteasome system (UPS) mediates the degradation of short-lived proteins and has been indicated as an important mechanism in disuse atrophy (Malavaki et al., 2015). Two major muscle-specific ubiquitin ligases that contribute to the ubiquitination process include muscle RING-finger protein-1 (MuRF-1) and atrogin-1/MAFbx. For instance, knee immobilization in humans significantly triggers the expression of MuRF-1 and atrogin-1 after 14 days (Jones et al., 2004). Animal studies have also mentioned the remarkable upregulation in the mRNA and protein expression of MuRF-1 and atrogin-1 under disuse condition (Watanabe et al., 2016; Ito et al., 2017). Another process contributing to the loss of muscle mass under disuse condition is inflammation. Induction of nuclear factor kappa B (NF-κB) activity is known to play a significant role in disuse muscle atrophy (Hunter et al., 2002). Upregulation in the mRNA levels of proinflammatory cytokines such as tumor necrosis factor (TNF)-α, interleukin (IL)-1β, and IL-6 have been observed after 14 days of immobilization (Kim et al., 2015). In addition, apoptosis is shown to be involved in the development of disuse muscle atrophy (Yoshihara et al., 2017; Mi Gong et al., 2018).

Glucagon-like peptide-1 (GLP-1), an incretin hormone produced by the intestinal mucosa, is secreted after meal consumption and stimulates insulin secretion (Hinnen, 2017). The effect of GLP-1 receptor agonist is not limited to the pancreas, as its receptor is known to be expressed in other organs such as the brain, kidney, heart, and skeletal muscle (Thompson and Kanamarlapudi, 2013). Studies have demonstrated that the exposure to exendin-4 (Ex-4) could increase glucose uptake by stimulating the 5′-AMP-activated protein kinase (AMPK) signaling pathway in rat L6 myotubes (Andreozzi et al., 2016). In addition, Ex-4 increased oxygen consumption and thermogenic gene expression in C2C12 myotubes (Choung et al., 2017) and was recently reported to exert therapeutic effects in dexamethasone-induced muscle atrophy (Hong et al., 2019).

In the present study, we investigated whether dulaglutide, a long-acting GLP-1 receptor agonist, exhibits any beneficial effects on disuse muscle atrophy and evaluated the underlying mechanism. Our results show that dulaglutide treatment attenuated muscle wasting and recovered muscle strength by inhibiting inflammation and apoptosis through the induction of heat shock protein 72 (Hsp72) expression in disuse condition.

## Materials and Methods

### Animals

Ten-week-old C57BL/6N male mice were obtained from Orient Bio (Seongnam-si, Kyunggido, Korea) and subjected to 1 week of adaptation before the study. All animal experiments were performed in compliance with the ethical requirements of the Laboratory Animal Research Center, College of Pharmacy, Gachon University. The experimental protocol was approved by the Gachon University Institutional Animal Care and Use Committee (GIACUC-R2018012). Mice were divided into four groups, including control + vehicle (CV), control + dulaglutide (CD), immobilization + vehicle (IV), and immobilization + dulaglutide (ID) groups. Mice from IV and ID groups were immobilized with spiral wire test to introduce disuse condition for 4 days, while those from the other groups were maintained under normal condition. After 4 days of immobilization, mice from CD and ID groups were subcutaneously injected with 600 µg/kg of phosphate-buffered saline (PBS)-diluted dulaglutide (Eli Lilly, IN, USA; 1.5 mg/0.5 ml). Other groups were injected with PBS. On the day dulaglutide injection, mice from CV and IV groups were fed same amount of food as consumed by dulaglutide-treated groups. These mice were maintained for additional 7 days. The steel wire was removed after day 10, and the grip strength was determined on day 11 to measure the muscle force before sacrifice. During the experiment, body weight and food intake were recorded around 10:00–11:00 AM daily.

### Immobilization Procedure

Mice from IV and ID groups were subjected to SWI model according to the Akiko Onda method (Onda et al., 2016). Mice were anesthetized with isoflurane (AbbVie, Berkshire, UK) and maintained in the supine position. Hair from hindlimb areas was shaved with a clipper and the ankle joints were taped in a bilateral plantar flexed position with medical bandage tape. A steel wire (2.0 m/m, Dongsung, Korea) was applied at the level of the L4–5 spine and coiled around the hip joints and both hindlimbs for bilateral SWI. Hindlimbs were fixed at a right angle to the trunk at the hip joint.

### Cell Culture

C2C12 cells (CRL-1772, ATCC^®^, USA) were cultured in Dulbecco's modified Eagle's medium (DMEM, LM001-05, Welgene, Korea) supplemented with 10% fetal bovine serum (PK004-07, Welgene, Korea), 100 IU/ml penicillin, and 0.1 mg/ml streptomycin (LS202-02, Welgene, Korea). C2C12 cells were seeded at 2.5 × 10^5^ cells/well in a six-well plate and incubated in a differentiation medium containing DMEM, 2% horse serum (26050-088, Gibco, UK), 100 IU/ml penicillin, and 0.1 mg/ml streptomycin for 5 days. To evaluate AMPK activity and Hsp72 protein level, the differentiated myotubes were treated with dulaglutide (1.5 µg/ml for 12 h). To evaluate AMPK activity, myotubes were treated with Ex-4 (≥ 97% purity, E7144, Sigma, USA) for 30 min and Hsp72 protein level was assessed after 3 h exposure to 20 nM Ex-4. To investigate whether the dulaglutide- or Ex-4- mediated increase in Hsp72 expression is regulated by AMPK activation, C2C12 myotubes were treated with compound C (≥ 98% purity, P5499, Sigma, USA), an AMPK inhibitor, at 20 µM for 1 h before exposure to dulaglutide (1.5 µg/ml for 3 h) or Ex-4 (20 nM for 30 min or 3 h).

### Grip Strength

After 10 days of treatment, hindlimb muscle strength was measured using a grip strength meter (BIO-G53, BIOSEB, FL, USA). For hindlimb strength analysis, an angled mesh assembly was applied. Mice were allowed to rest on the mesh until they could tightly grip the mesh by only two hindlimbs. The two hindlimbs were placed at least one-half of the way down the length of the mesh. The tail was pulled directly toward the tester and parallel to the mesh assembly with the same force. Grip strength was calculated as force divided by final body weight (g/g).

### Tissue Collection

At the end of experiment, tissues were rapidly excised, carefully dissected, and weighed. Isolated skeletal muscles were gastrocnemius (GA), soleus, extensor digitorum longus (EDL), tibialis anterior (TA), and quadriceps femoris (QD). Muscle samples were stored at −80°C until analyses.

### Histology

GA muscles were fixed in 10% neutral buffered formalin and embedded in paraffin. These paraffin blocks were cut into 5-μm-thick sections and stained with hematoxylin (30002, MUTO PURE CHEMICALS CO., LTD., Japan) and eosin (HT110132, Sigma, USA) (H&E). The H&E-stained sections were used for CSA analyses and examined (200× magnification) under a confocal microscope (Nikon Intensilight C-HGFI, Japan) using NIS-element AR 4.00.00 software. The myofiber CSAs were analyzed with ImageJ program.

### Real-Time Polymerase Chain Reaction (PCR) Analysis

Total RNA was isolated from the GA muscle using RNAiso Plus (9108, TAKARA, Japan) following the manufacturer's protocol. Complementary DNA (cDNA) was synthesized from 2 μg of total RNA with the PrimeScript 1st-strand cDNA synthesis kit (6110A, TAKARA, Japan). Real-time quantitative PCR (RT-qPCR) was performed using a reaction mixture comprising SYBR Green master mix (RR820A, TAKARA, Japan). Results were calculated using the 2−ΔΔCT relative quantification method and normalized to the glyceraldehyde 3-phosphate dehydrogenase (GAPDH) gene expression level. The sequences of the primer pairs are shown in **Table 1**.

**Table 1:** Primer sets used for quantitative PCR analyses.

No.	Primer	Sense	Anti-sense
1	MuRF-1	AGG ACT CCT GCA GAG TGA CCA A	TTC TCG TCC AGG ATG GCG TA
2	Atrogin-1	GCA AAC ACT GCC ACA TTC TCT C	CTT GAG GGG AAA GTG AGA CG
3	TNFα	CCA ACG GCA TGG ATC TCA AAG ACA	AGA TAG CAA ATC GGC TGA CGG TGT
4	IL-1β	CTA CAG GCT CCG AGA TGA ACA AC	TCC ATT GAG GTG GAG AGC TTT C
5	IL-6	TCC AGT TGC CTT CTT GGG ACT GAT	AGC CTC CGA CTT GTC AAG TGG TAT
6	GAPDH	CGA ACA TCA TCC CTG CAT CCA C	CCC AGT GAG CTT CCC GTT CA
7	MYH I	CCA AGG GCC TGA ATG AGG AG	GCA AAG GCT CCA GGT CTG AG
8	MYH IIa	AAG CGA AGA GTA AGG CTG TC	GTG ATT GCT TGC AAA GGA AC
9	MYH IIb	ACA AGC TGC GGG TGA AGA GC	CAG GAC AGT GAC AAA GAA CG

### Western Blot Analysis

Total proteins from GA muscle or C2C12 myotubes were isolated with mammalian protein extract buffer (GE Life Science, USA) containing protease inhibitor cocktail (P8340, Sigma, USA) and phosphatase inhibitors (P5726, P0044, Sigma, USA). The isolated proteins were separated by sodium dodecyl sulfate polyacrylamide gel electrophoresis. Membranes were incubated with the following primary antibodies: anti-β-actin (#4857, Cell Signaling Technology, USA), anti-MuRF-1 (ab172479, Abcam, USA), anti-MAFbx (sc166806, Santa Cruz Biotechnology, USA), anti-GDF8/myostatin (ab203076, Abcam), anti-MYH (B-5) (sc-376157, Santa Cruz Biotechnology), anti-Bax (#2772, Cell Signaling Technology, USA), anti-caspase-3 (#9662, Cell Signaling Technology, USA), anti- poly-ADP ribose polymerase (PARP; #9542, Cell Signaling Technology, USA), anti-NF-κB p105/p50 (#13586, Cell Signaling Technology, USA), anti-IκBα (#4814, Cell Signaling Technology, USA), anti-p-IκBα (#9246, Cell Signaling Technology, USA), anti-Hsp72 (sc-66048, Santa Cruz Biotechnology), anti-p-AMPKα (#2531, Cell Signaling Technology, USA), and anti-AMPKα (#2532, Cell Signaling Technology, USA). The target complex was detected by ChemidocTM XRS^+^ system with Image Lab™ software (Bio Rad, USA) and band intensity was quantified by Image Lab program. The expression of proteins of interest was normalized to that of β-actin for densitometry analysis.

### Statistical Analysis

Data are presented as the mean ± standard error of the means (S.E.M). Statistical analysis was performed using an unpaired parametric analysis of variance (ANOVA), followed by Fisher's protected least significant difference test for multiple groups or Student's t-test for two groups. A value of p < 0.05 was accepted as significant.

## Results

### Dulaglutide Treatment Improved Muscle Strength and Reduced Muscle Wasting in Disuse Condition

**Figure 1:**
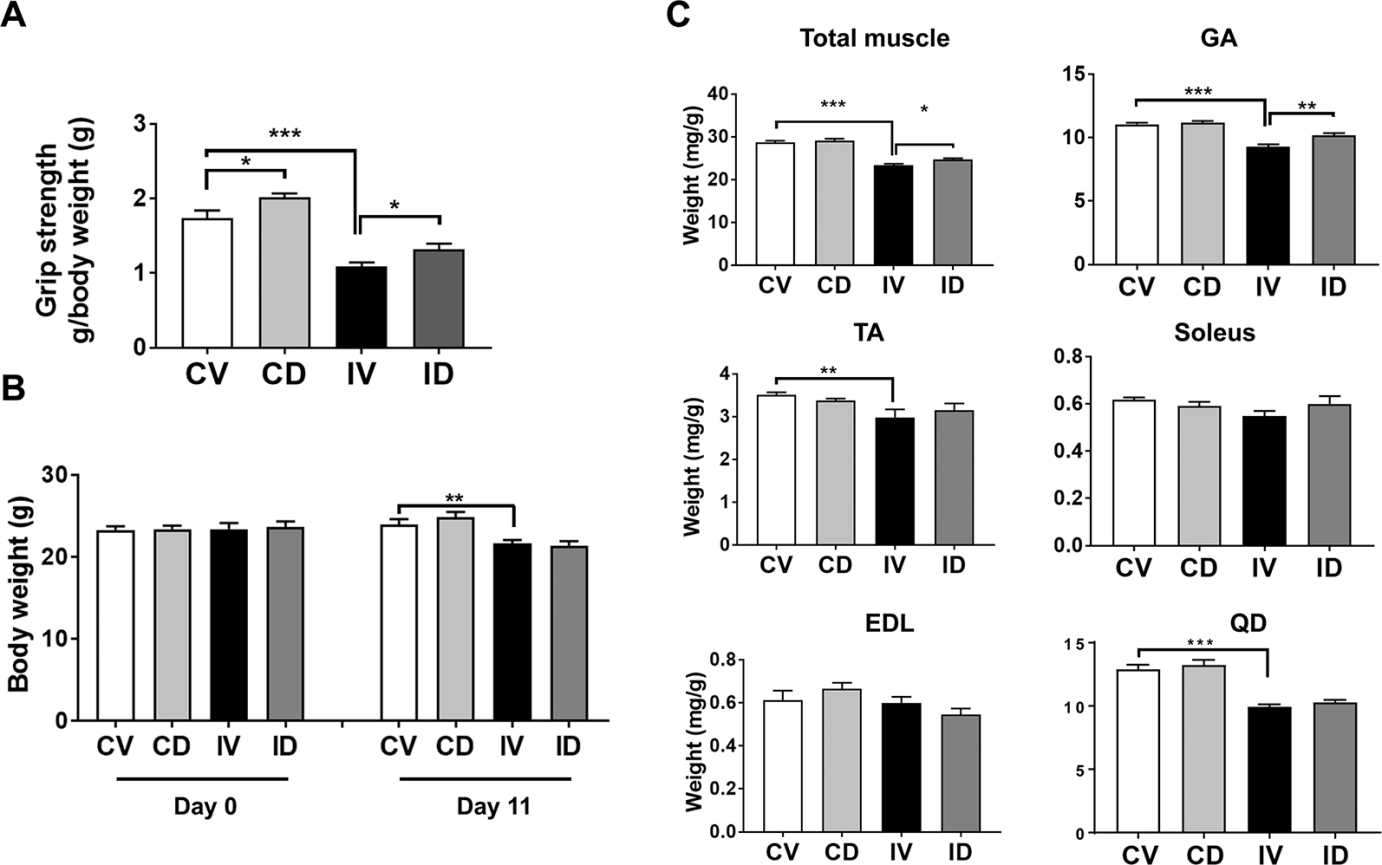
Dulaglutide improved muscle strength and attenuated muscle loss in disuse condition. Ten-week-old C57BL/6 male mice were subjected to spiral wire immobilization for 4 d and administrated dulaglutide (Trulicity, 600 µg/kg, subscutaneous). Mice were maintained for additional 7 d. **(A)** Grip strength was measured on day 11 and grip strength value was normalized to final body weight. **(B)** Body weights on day 0 and 11 were recorded. **(C)** The total muscle weight and weight of various muscle types, including gastrocnemius (GA), soleus, tibialis anterior (TA), extensor digitorum longus (EDL), and quadriceps (QD), were measured right after sacrifice and normalized to the final body weight. Data are shown as means ± S.E.M, n = 6–7/group; *p < 0.05, **p < 0.01, ***p < 0.001. CV, control + vehicle; CD, control + dulaglutide; IV, immobilization + vehicle, ID; immobilization + dulaglutide.

To evaluate whether GLP-1 receptor agonist exerts beneficial effects on muscle wasting in disuse condition, 10-week-old C57BL/6 mice were immobilized using spiral wire and treated with dulaglutide (Trulicity, 600 µg/kg, subcutaneous) at day 4 after immobilization and maintained for additional 7 days. Grip strength test result showed that the vehicle-treated immobilized mice (IV group) had a 40% (p < 0.01) decrease in muscle strength as compared with the control mice (CV group) (**Figure 1A**). Dulaglutide treatment (ID group) significantly improved the grip strength as compared with IV group (p < 0.05), suggestive of the dulaglutide-mediated improvement in muscle functions under disuse condition. Body weight decreased after immobilization, and dulaglutide treatment had no effect on body weight (**Figure 1B**). Total muscle weight was significantly lower for IV group than for CV group (p < 0.001) (**Figure 1C**), consistent with the decrease in the muscle mass during disuse condition reported in previous studies (Watanabe et al., 2016; Ito et al., 2017). The total muscle weight was significantly higher for ID group (p < 0.05) than for IV group. GA, TA, and QD muscle weights, but not soleus and EDL weights, were markedly lower for IV group than for CV group. Dulaglutide significantly increased (p < 0.01) GA muscle weight and showed a tendency to increase TA muscle and soleus muscle weights as compared with IV treatment. However, no change in EDL muscle weight was detected (**Figure 1C**).

### Dulaglutide Treatment Increased Muscle Fiber Size in Disuse-Induced Skeletal Muscle Atrophy

**Figure 2:**
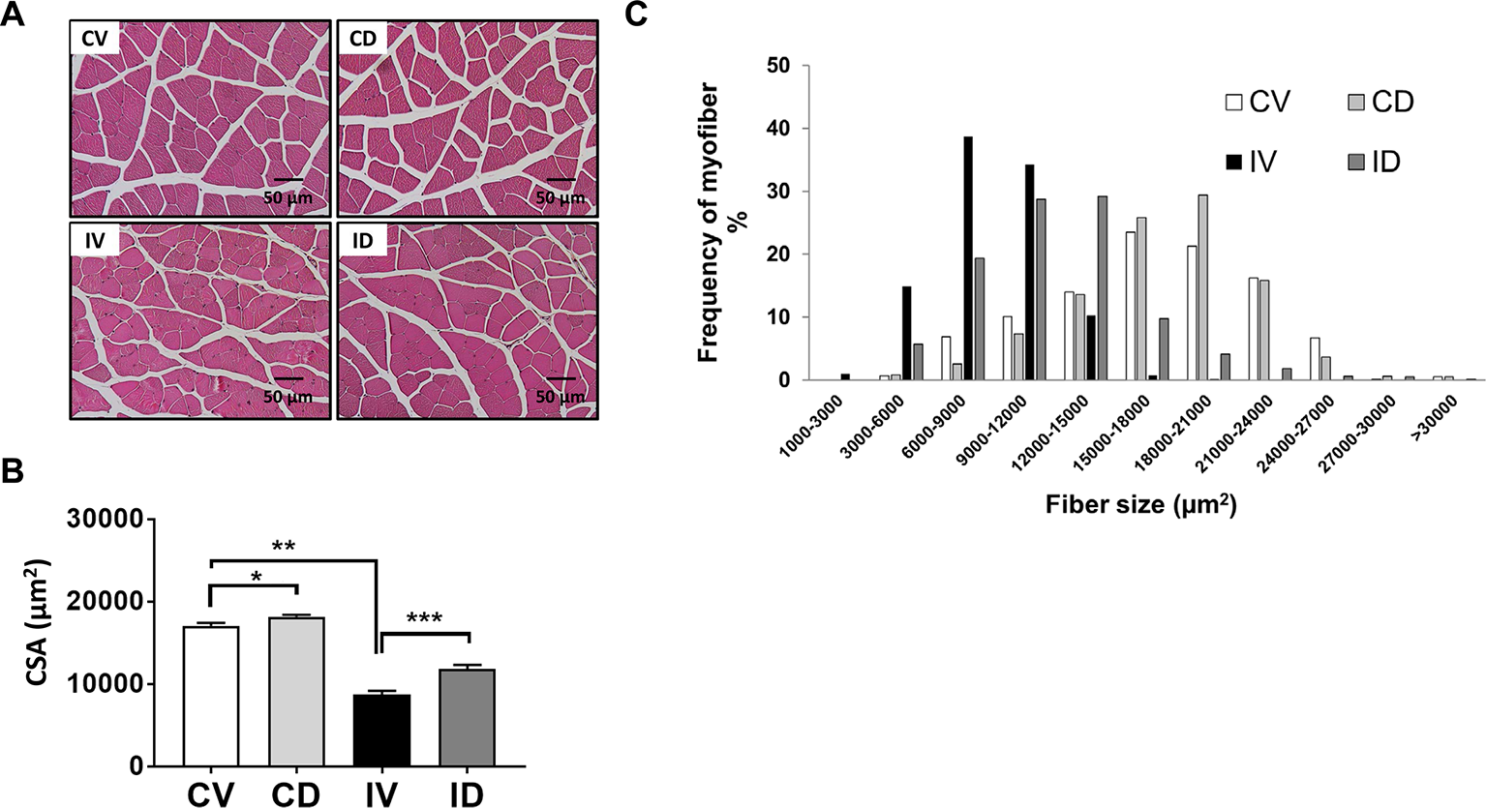
Dulaglutide treatment increased muscle fiber size in disuse-induced skeletal muscle atrophy. **(A)** GA muscle tissue sections were stained with H&E and examined under microscope. **(B)** The cross-sectional area (CSA) of muscle fiber was measured using ImageJ program and the average CSA is shown. **(C)** Distribution of myofiber size. Data are shown as means ± S.E.M, n = 5; *p < 0.05, **p < 0.01, ***p < 0.001 versus control + vehicle or immobilization + vehicle group. CV, control + vehicle; CD, control + dulaglutide; IV, immobilization + vehicle; ID, immobilization + dulaglutide.

The CSA of myofibers was found to be reduced in immobilization or hindlimb suspension-induced muscle atrophy (Caron et al., 2009; Ito et al., 2017). To characterize the internal structure of muscle fibers, GA muscle section was stained with H&E and myofiber size was analyzed. As shown in **Figures 2A, B**, approximately 50% reduction in CSA was observed for IV group as compared with CV group. Treatment with dulaglutide significantly increased the CSA. Dulaglutide treatment also significantly increased CSA in the control group (CD group) as compared with the CV group (p < 0.05). In terms of myofiber distribution, the predominant myofiber sizes were 15,000–18,000; 18,000–21,000; 6,000–9,000; and 12,000–15,000 µm^2^ for CV, CD, IV, and ID groups, respectively (**Figure 2C**). Taken together, the reduction in myofiber size as a result of disuse condition was significantly recovered by dulaglutide treatment.

### Dulaglutide Treatment Decreased the mRNA and Protein Expression Levels of Atrophic Genes in Disuse-Induced Muscle Atrophy

**Figure 3:**
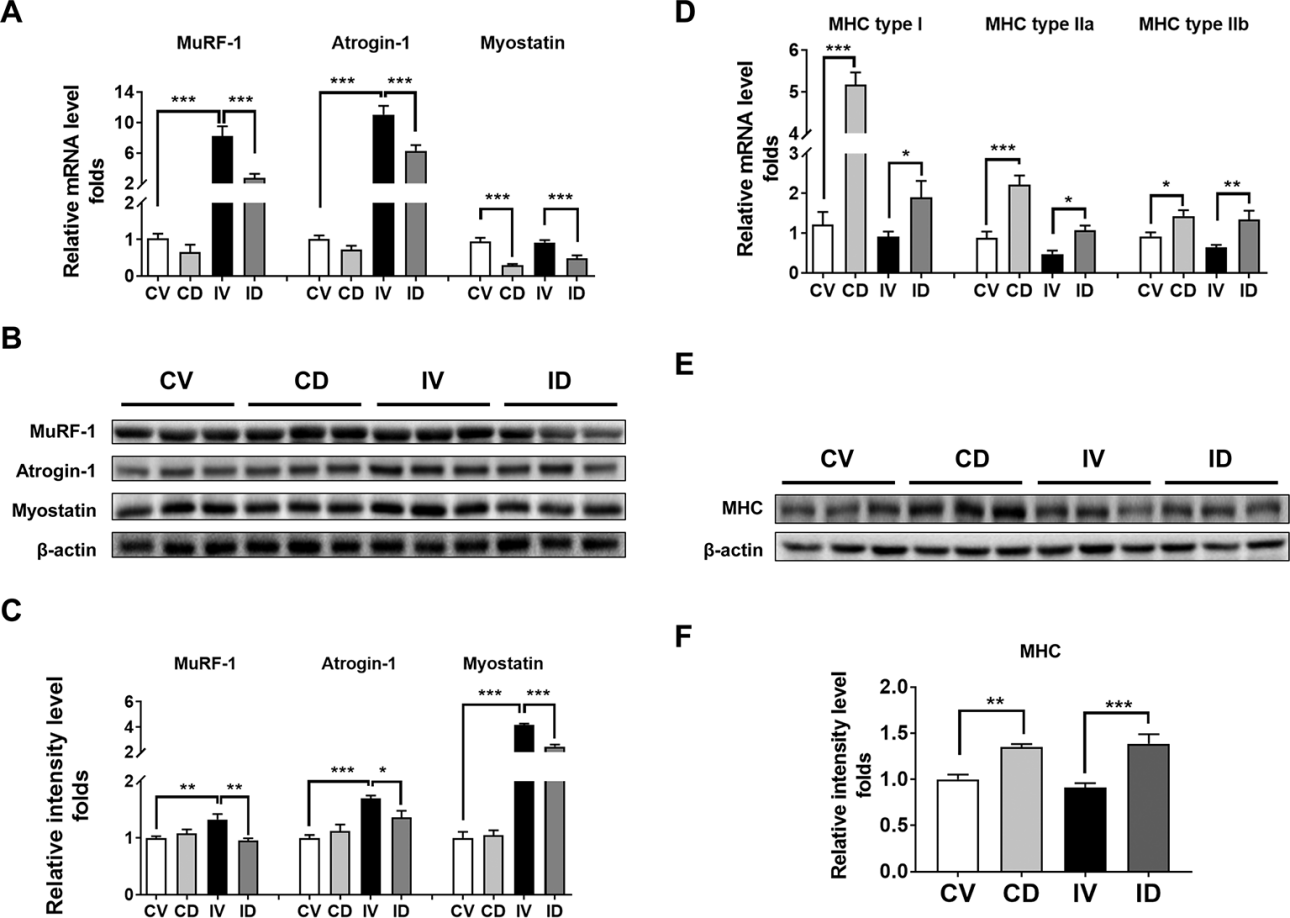
Dulaglutide treatment decreased the mRNA and protein expression levels of degradation-related genes under disuse-induced muscle atrophy. **(A)** The mRNA levels of the genes encoding MuRF-1, atrogin-1, and myostatin were analyzed with RT-qPCR in GA muscle tissue, n = 5. *GAPDH* mRNA served as an internal control. **(B)** Immunoblotting and **(C)** quantification analyses of MuRF-1, atrogin-1, and myostatin protein expression in GA muscle. Beta-actin was used as a loading control to ensure equal protein loading, n = 3. **(D)** The mRNA expression of myosin heavy chain isoforms, including myosin heavy chain type I, type IIa, and type IIb, was evaluated with RT-qPCR, n = 5. *GAPDH* mRNA was used as an internal control. **(E)** Immunoblotting and **(F)** quantification analyses of myosin heavy chain protein expression in GA muscle. Beta-actin was used as a loading control, n = 5. Data are shown as mean ± S.E.M. *p < 0.05, **p < 0.01, ***p < 0.001 as compared with control + vehicle or immobilization + vehicle group. CV, control + vehicle; CD, control + dulaglutide; IV, immobilization + vehicle; ID, immobilization + dulaglutide.

The mRNA and protein expression levels of MuRF-1 and atrogin-1 are known to be upregulated in disuse models (Watanabe et al., 2016; Ito et al., 2017). To investigate whether dulaglutide treatment could inhibit the expression of MuRF-1 and atrogin-1 in disuse condition, we examined the mRNA and protein levels of MuRF-1 and atrogin-1 in the GA muscle of dulaglutide-treated mice (ID group). Both mRNA and protein levels of these markers increased in disuse condition (IV group), and dulaglutide treatment (ID group) attenuated the increase in these levels (**Figures 3A–C**). Myostatin is a negative regulator of skeletal muscle growth and the knockdown of myostatin expression could prevent muscle wasting after 14 days of casting (Murphy et al., 2011). Both myostatin mRNA and protein levels significantly decreased after dulaglutide treatment (**Figures 3A–C**). Thus, dulaglutide intervention downregulated the expression of myostatin and the proteins involved in protein degradation, thereby contributing to the attenuation of the loss of muscle proteins in disuse condition. We examined the expression of myosin heavy chain (MHC), a motor protein of the muscle filament. Treatment with dulaglutide increased the mRNA level of MHC, including MHC type I, MHC type IIa, and type IIb (**Figure 3D**) as well as MHC protein expression (**Figures 3E, F**).

### Dulaglutide Treatment Reduced the Expression of Inflammatory Molecules in Disuse-Induced Muscle Atrophy

**Figure 4:**
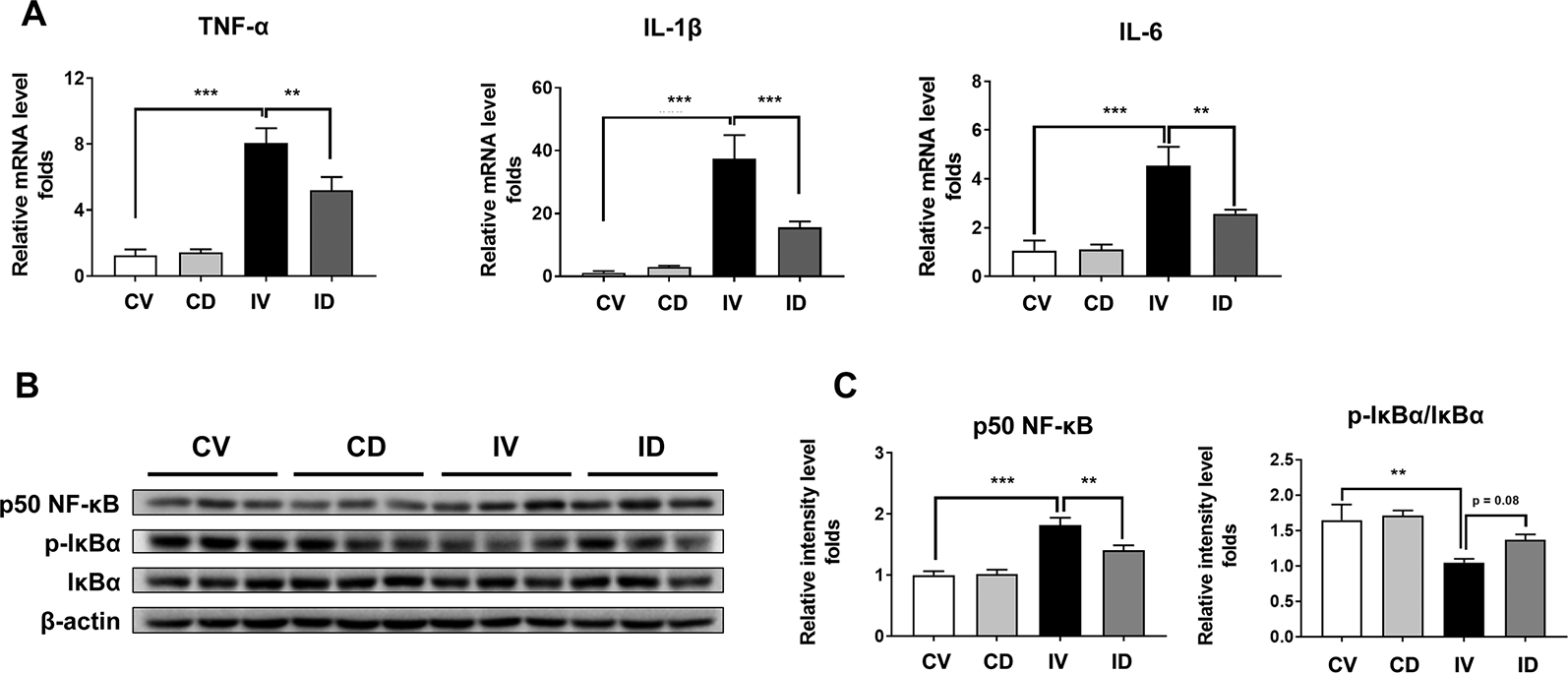
Dulaglutide treatment reduced the expression of proinflammatory cytokines and p50 NF-κB in disuse-induced muscle atrophy. **(A)** The mRNA expression levels of TNF-α, IL-1β, and IL-6 were analyzed with RT-qPCR in GA muscle tissue. *GAPDH* mRNA was used as an internal control. **(B)** Immunoblotting and **(C)** quantification analyses of p50 NF-κB and p-IκBα protein expression in GA muscle. Beta-actin was used as a loading control. Data are shown as mean ± S.E.M, n = 5; **p < 0.01 or ***p < 0.001 as compared with control + vehicle or disuse + vehicle group. CV, control + vehicle; CD, control + dulaglutide; IV, immobilization + vehicle; ID, immobilization + dulaglutide.

Inflammation contributes to muscle loss in disuse condition (Hunter et al., 2002). The mRNA levels of *TNF-α*, *IL-1β*, and *IL-6* were upregulated by up to 8-fold (p < 0.001), 30-fold (p < 0.001), and 4-fold (p < 0.01), respectively, in disuse condition as compared with that under normal conditions (CV group) (**Figure 4A**). However, the mRNA levels of these pro-inflammatory cytokines were significantly decreased after dulaglutide treatment. In addition, disuse condition was shown to induce the expression of p50 NF-κB and activate NF-κB signaling in the skeletal muscle (Hunter et al., 2002). We also found that the protein expression of p50 NF-κB significantly increased in immobilized mice (IV group) and the expression of p-IκBα, an inhibitor of NF-κB, decreased in the muscle of immobilized mice. The expression level of p50 NF-κB protein was significantly lower in dulaglutide-treated group (ID group) than in the vehicle-treated group (IV group). Furthermore, the protein level of phospho-IκBα tended to be restored after dulaglutide treatment (**Figures 4B, C**).

### Dulaglutide Treatment Attenuated the Expression of Apoptosis-Related Proteins in Disuse-Induced Muscle Atrophy

**Figure 5:**
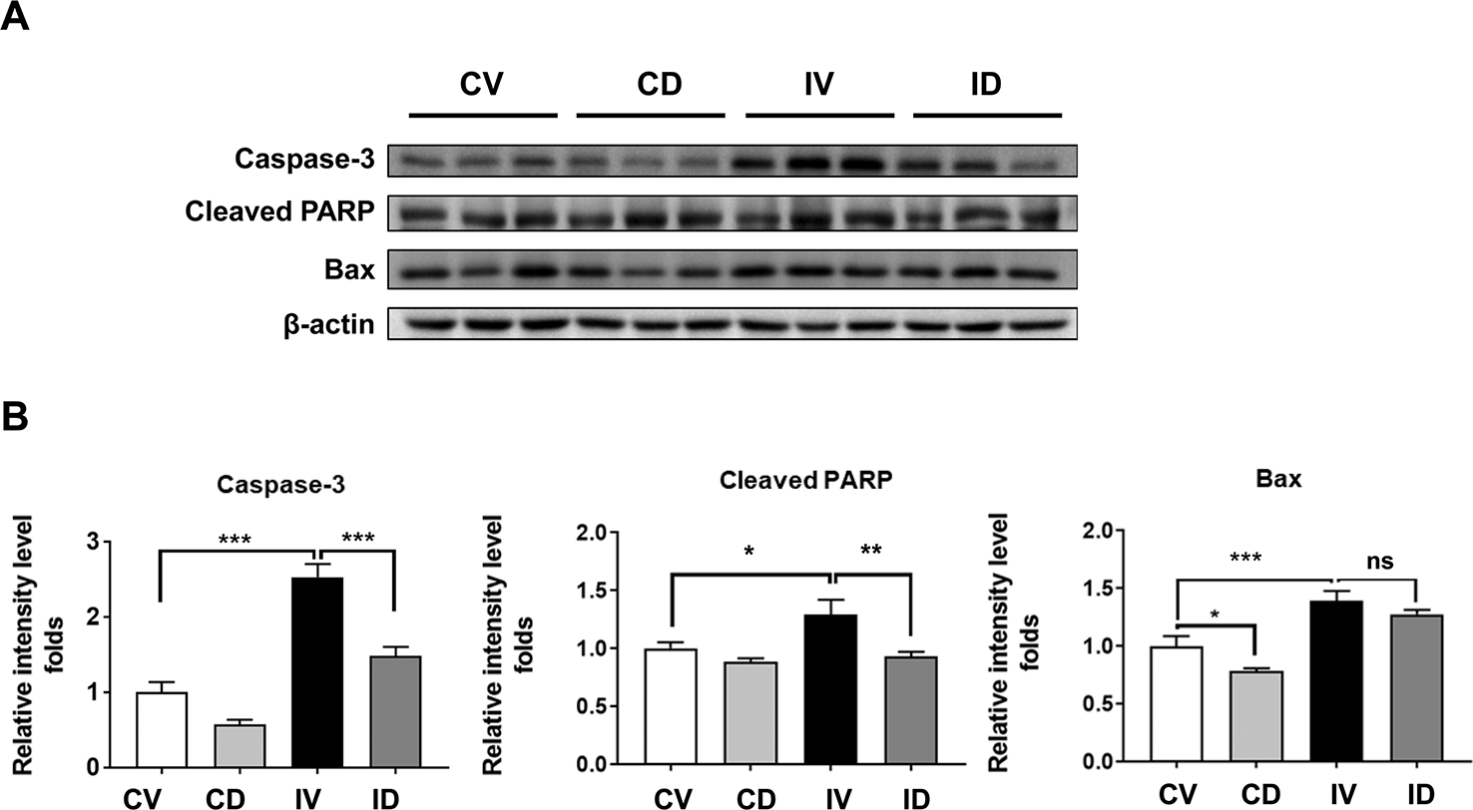
Dulaglutide prevents apoptosis in disuse condition. **(A)** Immunoblotting and **(B)** quantification analyses of caspase-3, cleaved PARP, and Bax proteins in GA muscle. Beta-actin was used as the loading control. Data are showed as mean ± S.E.M, n = 5; *p < 0.05, **p < 0.01, ***p < 0.001. CV, control + vehicle; CD, control + dulaglutide; IV, immobilization + vehicle; ID, immobilization + dulaglutide; ns, not significant.

Apoptosis is a key pathway involved in disuse-induced muscle atrophy. Apoptotic markers such as caspase-3, cleaved PARP, and Bax were reported to be elevated in disuse condition (Yoshihara et al., 2017; Mi Gong et al., 2018; Zhang et al., 2018). We also observed a significant increase in the levels of caspase-3, cleaved PARP, and Bax proteins after immobilization (**Figures 5A, B**). Treatment with dulaglutide, on the other hand, could significantly decrease the expression of caspase-3 and cleaved PARP as compared with the vehicle treatment in immobilized mice. Although the expression of Bax protein decreased, the effect was not significant (**Figures 5A, B**).

### Dulaglutide Treatment Increased the Expression of Hsp72 Through AMPK Signaling

**Figure 6:**
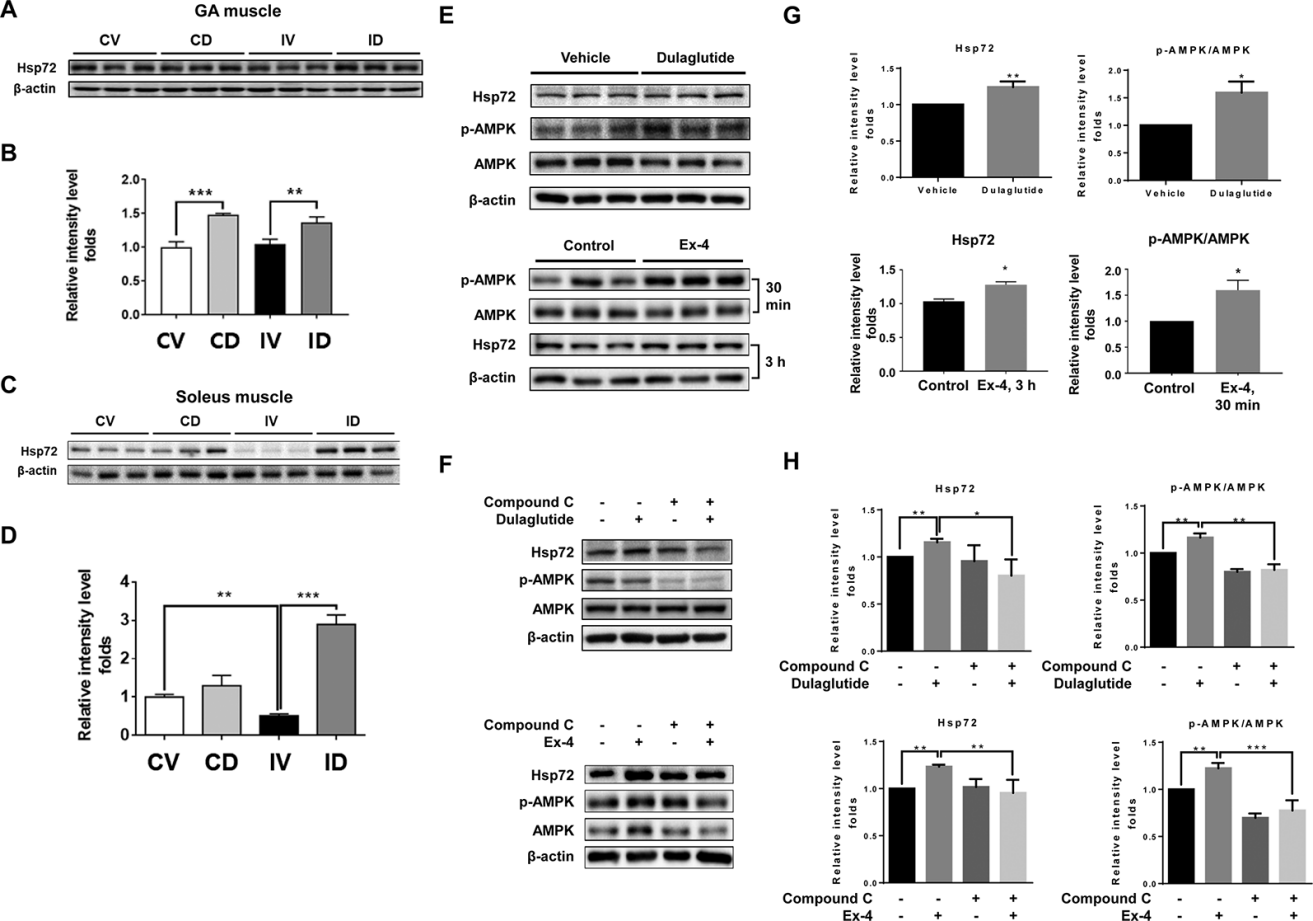
GLP-1 receptor agonist treatment increased Hsp72 protein expression through the regulation of AMPK signaling. **(A)** Immunoblotting and **(B)** quantification analyses of Hsp72 protein expression in GA muscle. **(C)** Immunoblotting and **(D)** quantification analyses of Hsp72 protein expression in soleus muscle. Beta-actin was used as a loading control. Data are shown as mean ± S.E.M, n = 5; *p < 0.05, **p < 0.01, ***p < 0.001. CV, control + vehicle; CD, control + dulaglutide; IV, immobilization + vehicle; ID, immobilization + dulaglutide. **(E)** Immunoblotting and **(G)** quantification analyses of Hsp72 and p-AMPK proteins in C2C12 myotubes treated with dulaglutide (1.5 µg/ml, 12 h) or Ex-4 (20 nM, 30 min or 3 h). **(F)** Immunoblotting and **(H)** quantification analysis of Hsp72 and p-AMPK proteins in C2C12 myotubes pre-treated with compound C at 20 µM for 1 h and then exposed to dulaglutide (1.5 µg/ml, 3 h) or Ex-4 (20 nM, 3 h). Beta-actin was used as the loading control. Data are shown as mean ± S.E.M, n = 3; *p < 0.05, **p < 0.01.

Hsp72 has been reported to inhibit muscle degradation and inflammation in the skeletal muscle and its overexpression could protect the skeletal muscle from atrophy following immobilization (Thakur Savant et al., 2018). Hsp72 protein expression level was unchanged in the GA muscle but significantly decreased in the soleus muscle after immobilization (**Figures 6A–D**). Dulaglutide treatment (ID group) significantly increased Hsp72 protein level in both GA (p < 0.01) and soleus (p < 0.001) muscles as compared with the vehicle treatment (IV group) after immobilization (**Figures 6B, D**). Dulaglutide also increased Hsp72 expression in the GA muscle of the non-immobilized control mice (**Figures 6A, B**). Hsp72 expression is regulated by sirtuin 1 (SIRT1) (Wang et al., 2017), and GLP-1 receptor agonist could activate SIRT-1 through the induction of AMPK signaling (Lin and Huang, 2016). Therefore, we investigated the effect of dulaglutide or Ex-4 on Hsp72 and p-AMPK expression in C2C12 myotubes *in vitro*. We found that dulaglutide or Ex-4 treatment significantly increased Hsp72 and p-AMPK protein levels (p < 0.05) (**Figures 6E, G**), and this effect was absent following treatment with the AMPK inhibitor, compound C (**Figures 6F, H**). These results indicate that GLP-1 receptor agonist, dulaglutide, and Ex-4, increases Hsp72 protein expression *via* AMPK activation.

## Discussion

The skeletal muscle plays a vital role in our body and performs multiple functions such as generation of force and power, maintenance of posture, production of movement, and contribution to basal energy metabolism (Frontera and Ochala, 2015). Long-time bed rest or physical inactivity causes muscle atrophy, which is a highly disabling and prevalent condition (Jackman and Kandarian, 2004). Overcoming disuse-induced skeletal muscle atrophy is still a major challenge, as therapeutic agents to treat this condition are limited. Decrease in muscle strength, loss in muscle mass, and reduction in muscle fibers are common during disuse time (Jaspers and Tischler, 1984). In addition, the activation of UPS, the main regulator of muscle degradation, has been reported in immobilization models (Onda et al., 2016). Other key processes such as inflammation and apoptosis are also known to be activated during inactivity period (Hunter et al., 2002; Yoshihara et al., 2017).

GLP-1 receptor agonists have been used to treat diabetes (Arnés et al., 2009). Aside from the blood glucose-lowering effects, GLP-1 receptor agonists also exert beneficial effects on the skeletal muscle by increasing glucose uptake (Thompson and Kanamarlapudi, 2013), fat oxidation, and thermogenic gene expression (Choung et al., 2017). In addition, GLP-1 receptor agonist, Ex-4, imparts therapeutic effects in muscle atrophy induced by dexamethasone (Hong et al., 2019). In the present study, we investigated the effect of dulaglutide, a GLP-1 receptor agonist, on disuse-induced muscle atrophy and evaluated the underlying mechanisms.

As GLP-1 receptor agonists reduce food intake (Ronveaux et al., 2014; Wan et al., 2017), the same amount of food as that consumed by dulaglutide-treated group was provided to the control vehicle-treated group. We examined body weight changes and found that immobilization significantly reduced body weight, and that dulaglutide treatment had no effect on body weight (**Figure 1B**). Decreased muscle strength is a diagnostic feature of muscle atrophy (Khan et al., 2013). Muscle strength decreased in mice following 10 days of immobilization (**Figure 1B**), contradicting the results of a previous report (Khan et al., 2013). Dulaglutide treatment showed stronger grip strength in immobilized mice than in vehicle-treated mice and recovered total muscle mass in mice subjected to disuse condition (**Figure 1C**). In a rodent immobilization model, the loss in extensor muscles of the ankle such as GA muscle was higher than that in the flexor muscles (TA and EDL) (Bodine, 2013). Here, we reported a significant reduction in GA, TA, and QD muscle weights following 10 days of immobilization. In particular, dulaglutide injection significantly increased GA muscle weight; we chose the GA muscle for further investigation. Mean CSA of the muscle decreased upon immobilization as previously reported (Caron et al., 2009; Ito et al., 2017) and dulaglutide treatment restored the CSA. Furthermore, the size of the predominant myofiber was larger in the dulaglutide-treated mice than in the vehicle-treated mice (**Figure 2C**). These results indicate that dulaglutide attenuated muscle wasting and increased muscle strength in disuse condition.

Skeletal muscle atrophy results from the imbalance between protein synthesis and degradation. UPS activation is one of the key processes contributing to the loss of the muscle during disuse condition, as it degrades the muscle protein (Malavaki et al., 2015). MuRF-1 and atrogin-1 play important roles in the degradation of contractile proteins (Malavaki et al., 2015). Upregulation in the mRNA levels of MuRF-1 and atrogin-1 was observed in various disuse atrophy models (Onda et al., 2011; Watanabe et al., 2016). We also found that the mRNA as well as the protein expression of MuRF-1 and atrogin-1 were significantly upregulated upon immobilization and that dulaglutide treatment ameliorated this effect (**Figures 3A–C**). The protein expression of myostatin, a negative regulator of skeletal muscle growth and development, was markedly induced following immobilization (**Figures 3B, C**). The mRNA level of myostatin was not different between the control and immobilization group. Myostatin mRNA level could peak at day 3 after the introduction of disuse condition and declined thereafter in later stages (Shao et al., 2007). Therefore, the increase in myostatin mRNA may not be observed at 10 days after immobilization, the time point of sampling, whereas myostatin protein level peaked around 7 days following disuse (Shao et al., 2007). Dulaglutide treatment significantly decreased myostatin mRNA and protein levels in immobilized mice. Taken together, dulaglutide may decrease myostatin expression and consequently reduce MuRF-1 and atrogin-1 activities, thereby contributing to the attenuation of the muscle degradation process under disuse condition.

Disuse-induced skeletal muscle atrophy is closely related to inflammatory process (Hunter et al., 2002). In addition, GLP-1-based therapies have been shown to exert anti-inflammatory effects in chronic inflammatory diseases (Kim et al., 2017). We evaluated the expression of inflammatory cytokines and found that the mRNA levels of *TNF-α*, *IL-1β*, and *IL-6* in the GA muscle were upregulated following 10 days from immobilization and that dulaglutide treatment inhibited this increase (**Figure 4A**). NF-κB activation is important for the induction of inflammatory cytokines, while p50 NF-κB, not p65, is activated during disuse condition (Hunter et al., 2002). IκBα is an inhibitory factor for NF-κB activation (Yamamoto and Gaynor, 2004). We found that p50 NF-κB level increased in immobilized mice while dulaglutide treatment ameliorated this effect. We also examined the expression of p-IκBα, a negative regulator (Yamamoto and Gaynor, 2004), and found that its expression was downregulated in immobilized mice and that dulaglutide treatment restored the levels (**Figures 4B, C**). These results suggest that GLP-1 receptor agonist may inhibit p-IκBα degradation and decrease p50 NF-κB, thereby contributing to the amelioration of inflammation during disuse conditions in the skeletal muscle.

Apoptosis is known to contribute to disuse-induced skeletal muscle atrophy (Yoshihara et al., 2017; Mi Gong et al., 2018; Zhang et al., 2018). In addition, GLP-1 receptor agonists have been known to have antiapoptotic effect on various cell lines such as beta cell, cardiomyocyte, and neural cell (Li et al., 2010; Ying et al., 2015; Chang et al., 2016). Therefore, we investigated the effects on apoptosis on the skeletal muscle in dulaglutide-treated mice under immobilization condition and found that dulaglutide treatment successfully reduced the induction caspase-3 and cleaved PARP proteins (**Figure 5**). The expression of Bax protein showed the decreased tendency by treatment with dulaglutide in immobilization condition, although not significant. These results indicate that dulaglutide protects the skeletal muscle against apoptosis and contributes to amelioration of muscle atrophy in disuse condition.

Hsp72 inhibits apoptosis (Clemons et al., 2005) and its overexpression decreases NF-κB activation through an increase in IκBα level (Sheppard et al., 2014; Thakur Savant et al., 2018). In addition, Hsp72 level is significantly downregulated in the muscle following immobilization (Senf et al., 2008). In the present study, we found reduction in Hsp72 level in the soleus muscle but not in the GA muscle of immobilized mice (**Figure 6**). This observation is probably attributed to the fact that Hsp72 mainly exists in slow type I fibers (Ogata et al., 2003), which are low in the GA muscle tissue. Dulaglutide significantly upregulated the expression of Hsp72 protein in both GA and soleus muscles. As SIRT1 is an upstream target of Hsp72 (Wang et al., 2017) and the induction of SIRT1 expression in the skeletal muscle is related to the activation of AMPK molecule (Lin and Huang, 2016), we hypothesized that the dulaglutide-mediated increase in Hsp72 expression level in the skeletal muscle may be mediated by AMPK activation. The results observed with AMPK inhibitor treatment show that the increase in Hsp72 protein expression after exposure to dulaglutide or Ex-4, GLP-1 receptor agonist, in C2C12 myotubes was regulated by AMPK (**Figures 6F, H**). GLP-1 receptor agonist increased Hsp72 expression level *via* AMPK activation that inhibited inflammation and apoptosis.

In conclusion, we demonstrate that treatment with dulaglutide, a GLP-1 receptor agonist, could recover muscle strength, muscle mass, and muscle fiber size, which were reduced during immobilization. Dulaglutide treatment attenuated the induction of atrophic genes, such as those encoding MuRF-1, atrogin-1, and myostatin, and enhanced MHC expression. In addition, dulaglutide treatment inhibited the expression of inflammatory cytokines and apoptotic genes through the induction of heat shock protein 72 (Hsp72) expression *via* AMPK activation, contributing to the amelioration of disuse-induced muscle atrophy.

## Data Availability Statement

The raw data supporting the conclusions of this article will be made available by the authors, without undue reservation, to any qualified researcher.

## Ethics Statement

The animal study was reviewed and approved by GIACUC-R2018012.

## Author Contributions

The authors’ responsibilities were as follows—H-SJ conceived and designed the study. HC and TN contributed to the design of the study and performed the experiments. HC, TN, and H-SJ wrote the manuscript. H-SJ critically revised the manuscript. All authors approved the final version of the manuscript.

## Funding

This study was supported by a grant from the Korea Health Technology R&D Project through the Korea Health Industry Development Institute (KHIDI), funded by the Ministry of Health & Welfare, Republic of Korea (grant number: HI14C1135), the Basic Science Research Program through the National Research Foundation of Korea (NRF), funded by the Ministry of Education (NRF-2017R1A6A3A11033316).

## Conflict of Interest

The authors declare that the research was conducted in the absence of any commercial or financial relationships that could be construed as a potential conflict of interest.
